# Breast cancer in kurdish women of northern Iraq: incidence, clinical stage, and case control analysis of parity and family risk

**DOI:** 10.1186/1472-6874-9-33

**Published:** 2009-12-11

**Authors:** Runnak A Majid, Hazha A Mohammed, Heshu M Saeed, Banaz M Safar, Rekawt M Rashid, Michael D Hughson

**Affiliations:** 1 Department of Pathology, Shorsh General Hospital, Sulaimaniyah, Iraq; 2 Division of Oncology, Hewa Hematology and Oncology Hospital, Sulaimaniyah, Iraq; 3 Division of Epidemiology and Statistics, Directorate of Health, Sulaimaniyah, Iraq; 4 Office of the Director of Health, Sulaimaniyah Governate, Sulaimaniyah, Iraq

## Abstract

**Background:**

Breast cancer in the Middle-East occurs in relatively young women and frequently presents as advanced disease. A protective effect of multiparity is not apparent, and high familial risk is reported in some countries. This study investigates breast cancer rates and clinical stage related to age in the Kurdish region of Iraq and evaluates risk associated with parity and family history. Findings are compared with nearby countries and the West.

**Methods:**

Sulaimaniyah Directorate of Health records identified 539 women diagnosed with breast cancer during 2006-2008. Clinical survey forms were completed on 296 patients and on 254 age-matched controls. Age specific incidence rates were calculated from Directorate of Health population estimates.

**Results:**

Average patient age was 47.4 ± 11 years and 59.5% were pre-menopausal. Diagnosis was at clinical stage 1 for 4.1%, stage 2 for 43.5%, stage 3 for 26.0%, and stage 4 for 8.1% of patients. For 18.2%, stage was unknown. Annual breast cancer incidence rates per 100,000 women peaked at 168.9 at age 55 to 59 and declined to 57.3 at 60 and above. Patients had an average of 5.0 ± 3.3 children compared to 5.4 ± 3.5 for controls, *P *= 0.16. A first degree family member had breast cancer among 11.1% of patients and 2.1% of controls (*P *< 0.001) with > 50% of these patients and controls being ≥50 years old. No statistically significant relationship was found between tumor stage and age, *P *= 0.59.

**Conclusions:**

In Kurdish Iraq, breast cancer is predominantly a disease of pre-menopausal women having multiple pregnancies. For younger patients, breast cancer incidence was similar to the West and possibly higher than many Middle-Eastern countries, but unlike the West, the estimated rates declined markedly in the elderly. The familial breast cancer risk for both older and younger women was within the general population risk of Western countries. Clinical stages were advanced and indicated delays in diagnosis that were unrelated to patient age.

## Background

The incidence of breast cancer appears to be increasing world-wide [1]. The increase in Western countries is well documented and is paralleled by stable or declining breast cancer mortality. These divergent findings are presumed to be due to more effective therapies and to an early detection of small tumors [2]. In non-Western countries accurate epidemiologic data is difficult to collect, but breast cancer may also be increasing in less developed regions of the world [2].

In both the Middle-East and the West, carcinoma of the breast is the most common malignancy of women. In the West, there is a cultural tendency toward late marriages and limited childbearing. In this setting, multiparity and breast feeding are protective against breast cancers that are predominantly found after the menopause [2]. In the Middle-East, breast cancer is frequently seen during the childbearing years [2,3]. Studies on breast cancer rates and risk factors in the Middle-East are limited, but Egypt is thought to have the highest rates in the Middle-East with the increase being primarily between 30 to 60 years of age [2,4].

A case control study from Iran did not shown any relationship between parity and breast cancer among 286 patients whose average age at diagnosis in 1997-1998 was 47.5 ± 12.5 years [5,6]. The investigators did, however, find a significantly increased risk among women who were never married and among women with a positive family history of breast cancer. They also found that the great majority of patients were diagnosed with advanced disease, and that many patients had a significant delay between their recognition of symptoms and their first medical consultation for the condition.

In this initial study of breast cancer in the Kurdish region of northern Iraq, we investigated age specific cancer rates within Sulaimaniyah province. In an age-matched case control study, we evaluated the risks associated with reproductive history and family breast cancer history. We additionally analyzed the relationships between patient age and clinical stage as a means of assessing tumor behavior in older and younger women. The aims of the study were to compare breast cancer rates, the severity of disease, and risk associated with reproductive and family history with published investigations from other Middle-Eastern countries and from the United States.

## Methods

The Sulaimaniyah Directorate of Health serves as the ethics committee for Hewa Hospital. The Directorate of Health gave permission for the research, and patient interactions were conducted according to the Helsinki Accords. In 2005, the Directorate of Health established Hewa Hematology and Oncology Hospital as the central institution for collecting data on cancer patients and coordinating cancer care in the region. In the years 2006 through 2008, 539 women were reported as having a first diagnosis of breast cancer. Registry included age, place of residence, and the record of the histologic confirmation of diagnosis. Among the 539 women, 296 were treated at Hewa Hospital where detailed clinical records were available and patients could be interviewed during clinic visits. All 296 patients gave oral consent to be interviewed by RAM, HAM, or BMS. The remaining 243 patients were treated at other regional facilities. Additional records beside registration were not available at Hewa Hospital, and patients were not further contacted.

The interview used a structured clinical survey form that included age and menstrual status at the time of diagnosis, marital status, height, weight, number of children, age at first pregnancy, and family history of breast cancer with the relationships to affected family members. Height and weight were measured during the initial clinical examinations. The records of 242 patients contained an assignment of tumor stage at the time of diagnosis. For 54 patients, the extent of disease was unknown.

The questionnaires used for patients were also used for 254 control subjects attending general (non-obstetrical) ambulatory facilities unrelated to Hewa Hospital. Control subjects had no history of cancer. The numbers of control subjects were matched for the number of patients in each five year interval from 20 to 80 years old. The population and age distribution of male and female citizens of Sulaimaniyah Governate were obtained from the Division of Vital Statistics of the Directorate of Health.

Data were entered into an Excel worksheet and were analyzed with Stata software (Stata Corp., College Station, TX). Between groups comparisons were analyzed by t-tests if data met normality and equal variance tests and by Mann-Whitney rank-sum tests if they did not. Kolmogorov-Smirnov tests were applied to determine whether data were normally distributed. For the age grouped patients and controls, conditional logistic regression was used to analyze the observed risk of breast cancer (outcome) conferred by the predictors of marital status and number of children. The relationship between tumor stage and patient age was tested by linear regression and differences between groups for categorical values by chi-square tests. For all statistical procedures, *P *< 0.05 was considered statistically significant.

## Results

In 2006-2008, the following numbers of women were diagnosed with breast cancer: 188 patients in 2006, 205 patients in 2007, and 149 patients in 2008. The estimated Sulaimaniyah female population for 5 year age ranges from 20 though 75+ years and the annual age specific breast cancer incidence for the region are shown in Table 1. This is compared with published data for Israeli Arabs, Jordan, Egypt, and for the United States [2,3]. The Kurdish incidence rates exceeded those for Israeli Arab and Jordanian women and were similar to Egyptian women beginning at age 35-39. Kurdish rates reached a peak of 168.9 per 100,000 at 55-59 and then declined markedly to where they were less than half that for Jordanian, Israeli Arab, and Egyptian women at age 70-74 and approximately one-third of the rates for those regions at 75 years of age and above.

**Table 1:** Estimated age specific incidence (annual × 100,000) for breast cance in the Sulaimaniyah Governate and comparison with other Middle-Eastern countries and the United States [2,3].

age	Patients*	Population	Sulaimaniyah	Israeli Arabs	Jordan	Egypt	US
20-24 y	2	82,206	0.8	0.0	0.8	1.4	1.3

25-29 y	13	80,746	5.5	8.7	5.7	9.8	7.1

30-34 y	34	62,706	18.1	11.8	20.8	28.9	25.2

35-39 y	63	37,634	55.8	35.2	47.1	63.6	61.7

40-44 y	83	26,768	103.4	53.4	73.6	96.7	117.5

45-49 y	89	23,502	126.2	93.5	82.6	144.9	192.1

50-54 y	84	20,156	138.9	104.2	129.3	171.5	253.1

55-59 y	77	15,200	168.9	124.0	114.6	181.2	332.4

60-64 y	32	14,197	75.1	144.0	134.8	144.2	386.8

65-69 y	34	12,123	93.4	136.8	131.1	105.0	431.1

70-74 y	17	12,419	45.6	118.7	103.0	94.1	458.7

75+ y	11	15,970	22.9	96.4	77.6	99.6	458.7

*The number of women reported as having breast cancer in the Sulaimaniyah Governate during the 3 year period 2006-2008.

Population: estimates by age for Sulaimaniyah Governate.

Abbreviation: US, United States

The clinical characteristics of patients and case controls are shown in Table 2. The average age of breast cancer patients was 47.4 ± 11.0 years with a median age of 46 and the 25^th ^and 75^th ^percentiles being 39 and 55 years old respectively. The proportion of patients reporting that they were menstruating at the time of diagnosis and who were designated as pre-menopausal was 59.5%. There were no significant differences between breast cancer patients and controls in BMI, marital status, and number of pregnancies. The average BMI was in the overweight category, but the median BMI was just above normal weight at 26.7 kg/m^2 ^and the 25^th ^and 75^th ^percentiles were 24.4 and 30.3 kg/m^2 ^respectively. Breast cancer patients ≥50 years old had significantly fewer children than the same aged control subjects (*P *= 0.001), and breast cancer patients ≥50 years old were more often nulligravida than controls with this difference tending toward but not being significant. By conditional logistic regression, the independent variables of marital status (*P *= 0.76) and number of children (*P *= 0.08) were not significant predictors of breast cancer, although the number of children approached significance. The regression coefficient suggests a trend for fewer children being related to breast cancer (r = -0.063, 95%CI: -.0.132 to 0.006) with this effect being seen in subjects ≥50 years old (*P *= 0.03, r = -0.111, 95%CI: -0.210 to -0.012) but not < 50 years of age (*P *= 0.77, r = -0.015, 95%CI: -0.114 to 0.084).

**Table 2:** Clinical characteristics of patients at the time of diagnosis and of controls. Controls are matched with patients for age.

	Breast cancer n = 296	Controls n = 254	*P*
Age	47.4 ± 10.9	46.9 ± 12.7	0.65

Pre-menopausal	59.5%	55.9%	0.75

BMI	27.7 ± 5.0	27.1 ± 4.3	0.16

≥50 years old	39.5%	41.3%	0.84

Married	84.1%	91.7%	0.53

Never married ≥50 y	6.8%	5.7%	0.96

Nulligravida	13.9%	10.6%	0.38

Nulligravida ≥50 y	9.4%	2.8%	0.11

Children	5.0 ± 3.3	5.4 ± 3.5	0.16

Children, patients ≥50 y	6.1 ± 3.3	7.6 ± 3.3	0.001

History of mother or sister having breast cancer	11.1%	2.1%	< 0.001

History of mother or sister having breast cancer Patients ≥50 y	14.1%	3.1%	0.02

Values for age, BMI, and number of children are mean ± standard deviation.

Patients reported that a family member with any degree of relationship had breast cancer in 19.0% of cases compared to 7.1% of controls (OR 3.25, 95%CI: 1.82 to 5.79, *P *< 0.001). A first degree family member (mother or sister) had breast cancer among 11.1% of patients and 2.1% of controls (OR 5.85, 95%CI: 2.21 to15.46, *P *< 0.001). For subjects having an affected first degree family member, 14 out of 27 patients (51.9%) and 3 out of 5 controls were ≥50 years old. The odds ratio for patients having a first degree family member with breast cancer was similar for older and younger patients (≥50 years old, OR 5.21, 95%CI: 1.45 to 18.77, *P *= 0.01; < 50 years old, OR 6.89, 95%CI: 1.53 to 31.13, *P *= 0.01).

Table 3 shows the distribution of clinical stage among 296 surveyed patients. Just 4.1% of patients were diagnosed with stage 1 breast cancer; while 69.5% of patents had clinical stage 2 or stage 3 disease, and 8.1% had confirmed distant metastases. For patients with a designated stage, there was no significant relationship between tumor stage and age (r = -0.002, 95%CI: -0.011 to 0.006, *P *= 0.59) nor was the relationship significant when patients whose stage was unknown were included (r = -0.009, 95%CI: -0.022 to 0.003, *P *= 0.13).

**Table 3:** Distribution of clinical stages.

	All patients	≥50 years old
Clinical Stage		

Stage 1	12 (4.1%)	2 (1.7%)

Stage 2	129 (43.5%)	58 (49.6%)

Stage 3	77 (26.0%)	21 (17.9%)

Stage 4	24 (8.1%)	13 (11.1%)

Unknown	54 (18.2%)	23 (19.7%)

Total	296	117

## Discussion

In this study, the age specific annual incidence rates of breast cancer for women between 35 to 59 years of age were higher among Iraqi Kurds than for Israeli Arabs and Jordanians and were similar to Egyptians [2]. However at age 60, the Kurdish breast cancer rates fell abruptly and at 70 years of age and above they were less than half that of Jordanians, Israeli Arabs, or Egyptians.

The completeness of the registration of incident breast cancer patients is a concern. Beginning in 2005, the Directorate of Health centralized the registration of cancer patients at the Hewa regional Hematology and Oncology Hospital. Some patients have the financial means to seek private care outside of Iraq and never register. Other persons never register because they do not want further medical care once a diagnosis of cancer is made. Although there are no supporting data to substantiate the estimate, the proportion of non-registrants is thought to be 10 to 15%. This is an area that needs further investigation, and it is important to recognize that breast cancer rates throughout the Middle-East are very likely to be substantially underestimated compared to the West.

The fall in breast cancer rates for older Kurdish women could be due to age-selective non-referral for registration. Since 2005, there have been public efforts to promote breast cancer awareness. The low numbers of patients ≥60 years of age may be the result of these efforts not reaching or not being applied to the older population. On the other hand, the lower rates of breast cancer after age 60 could be the effect of multiparity actually reducing breast cancer development after the menopause.

Patient recognition and the clinical detection of breast tumors did not seem to be any better in older compared to younger women. Without regard to age, only 4.1% of patients had their breast cancers discovered as localized tumors; whereas, 69.5% were diagnosed at clinical stage 2 or 3 of the disease. Because of the lack of access to adequate radiologic procedures, stage 4 may have been under diagnosed at 8.1% of patients, and 18.2% of patients were referred to oncological services with insufficient information for a clinical stage to be assigned. In the United States, a contrasting majority of 61% of patients is diagnosed with stage 1 breast cancer, and in only 2% is stage unknown [3]. In all probability, the advanced stage at which tumors were found was the result of delayed diagnosis owing to a lack of adequate screening together with a cultural reluctance to seek earlier care. Nevertheless, on the basis of the similarity in clinical stage in older and younger women, age did not seem to influence whether patients took advantage of the care that was available.

These Iraqi breast cancer patients had an age distribution that was nearly the same as that seen in Iran, Egypt, Jordan, and among Israeli Arabs [2,5,6]. In all of these Mid-Eastern countries the average and median age at diagnosis is less than 50 and 25% of patients are younger than 41 years old. This compares with women in the United States where the median age at diagnosis is 61 years of age, 65% are 55 or older, and only 10.6% are younger than 44 years old [3].

Iraqi patients averaged more than five completed pregnancies before diagnosis. In the West where Caucasian women tend to have few children later in life, increased numbers of children reduce overall breast cancer risk but have little effect on tumor development before the menopause [7,8]. In contrast, African Americans on average have more children at an earlier age than Caucasians, and studies on African American women have shown a dual effect of childbearing on breast cancer risk [9]. While risk is reduced with increased parity for women ≥45 years of age, increased parity increases the risk of breast cancer in women less than 45 years old. We found that Sulaimaniyah patients ≥50 years old who were diagnosed with breast cancer were more often nulligravida and had significantly fewer children than controls of the same age, suggesting that reduced parity may increase breast cancer risk in older Kurdish women.

Age specific breast cancer incidence rates for Jordanian and Israeli Arab women lag 25 to 50% behind those in the United States until 40 to 44 years of age [2]. Thereafter, rates of breast cancer in the United States begin to double, and by age 65 they are more than 3 times the rates reported in the Middle-East including Egypt. The traditional culture of the Middle-East encouraged large families. The comparatively lower rates of breast cancer among older Middle-Eastern Arabs and Kurds could be a cohort effect due to increased childbearing having a protective effect on earlier generations of women.

There was an increased risk of breast cancer among Kurdish patients who had a positive family history compared to case controls. Nevertheless, the 11.1% of patients having a first degree relative with breast cancer was very similar to the proportions seen in two large prospective United States studies where 6.8% of the general population and 11.6% of breast cancer patients reported an affected mother or sister [10,11]. In Western countries, 5 to10% of all adult women have a first degree relative with breast cancer, and a positive family history for up to 17% of women for any degree of relationship is considered to be near the lifetime population risk [10-13].

Although 19% of Kurdish breast cancer patients gave a familial history of breast cancer, by Western standards this is not particularly high, and it is balanced by what appears to be a low familial breast cancer rate of 7.1% among case control subjects. In addition, the risks for both patients and controls were nearly equally distributed by age and did not seem to be a factor in a possible excess of cancers among pre-menopausal women.

Mammography is the principal tool for screening for breast cancer in Western medicine. The alternatives to mammography are clinical breast examinations and magnetic resonance imaging (MRI) with current evidence not justifying recommendations for either of these two methods as screening investigations [14,15]. Mammography significantly reduces breast cancer mortality for women aged 50 to 69 and to a lesser degree for women 40 to 49 years old [14]. The selective application of MRI may improve breast cancer detection in younger patients, and the American Cancer Society currently recommends MRI as an adjunct to screening mammography in high risk women ≥30 years old [16-18].

At the present time, there are no established breast cancer screening programs in the Kurdish region of Iraq. Recommendations are being formulated, but mammography is only available for patients referred to the University Hospitals in Sulaimaniyah, Erbil, and Dohuk and at a few private radiology facilities. It will take several years before meaningful mortality data will be available for our Kurdish population, but as screening is implemented, reductions in clinical stage might serve as a short-term measure of interventional programs.

## Conclusions

Breast Cancer in Kurdish women of Sulaimaniyah Iraq is currently diagnosed at advanced clinical stages with 60% of patients being under 50 years of age. Until approximately age 45, age specific incidence rates were similar to those of Egypt and the United States and were higher than reported for Israeli Arabs and Jordanians. These findings suggest that breast cancer risk for pre-menopausal Iraqi Kurds may be unusually high for a Middle-Eastern country. The cause of these seemingly higher pre-menopausal breast cancer rates could not be related to parity or to an excessive familial risk. Interventional programs need to be directed toward methods that will reduce clinical stage of disease in younger women, but the best approach to cost effective screening in our region is not readily apparent.

## Competing interests

The authors declare that they have no competing interests.

## Authors' contributions

RAM, HAM, and BMS contributed to data collection, data analysis, and the first draft of the manuscript. RAM was the principal contributor toward the study design. MDH and HMS contributed to study design and data analysis, and MDH wrote the final draft of the manuscript. RMR contributed to data collection and analysis and revision of the manuscript. All authors read and approved the final manuscript.

## Pre-publication history

The pre-publication history for this paper can be accessed here:

http://www.biomedcentral.com/1472-6874/9/33/prepub
